# The Clinical Assessment Study of the Hand (CAS-HA): a prospective study of musculoskeletal hand problems in the general population

**DOI:** 10.1186/1471-2474-8-85

**Published:** 2007-08-30

**Authors:** Helen Myers, Elaine Nicholls, June Handy, George Peat, Elaine Thomas, Rachel Duncan, Laurence Wood, Michelle Marshall, Catherine Tyson, Elaine Hay, Krysia Dziedzic

**Affiliations:** 1 Primary Care Musculoskeletal Research Centre, Keele University, Keele, Staffordshire, ST5 5BG, UK; 2 North Staffordshire Combined Healthcare NHS Trust, Stoke-on-Trent, Staffordshire, ST2 8LD, UK

## Abstract

**Background:**

Pain in the hand affects an estimated 12–21% of the population, and at older ages the hand is one of the most common sites of pain and osteoarthritis. The association between symptomatic hand osteoarthritis and disability in everyday life has not been studied in detail, although there is evidence that older people with hand problems suffer significant pain and disability. Despite the high prevalence of hand problems and the limitations they cause in older adults, little attention has been paid to the hand by health planners and policy makers. We plan to conduct a prospective, population-based, observational cohort study designed in parallel with our previously reported cohort study of knee pain, to describe the course of musculoskeletal hand problems in older adults and investigate the relative merits of different approaches to classification and defining prognosis.

**Methods/Design:**

All adults aged 50 years and over registered with two general practices in North Staffordshire will be invited to take part in a two-stage postal survey. Respondents to the survey who indicate that they have experienced hand pain or problems within the previous 12 months will be invited to attend a research clinic for a detailed assessment. This will consist of clinical interview, hand assessment, screening test of lower limb function, digital photography, plain x-rays, anthropometric measurement and brief self-complete questionnaire. All consenting clinic attenders will be followed up by (i) general practice medical record review, (ii) repeat postal questionnaire at 18-months, and (iii) repeat postal questionnaire at 3 years.

**Discussion:**

This paper describes the protocol for the Clinical Assessment Study of the Hand (CAS-HA), a prospective, population-based, observational cohort study of community-dwelling older adults with hand pain and hand problems based in North Staffordshire.

## Background

Musculoskeletal diseases have a major impact on the health of the population [1]. In adults aged 50 years and over osteoarthritis (OA) is the cause of the majority of musculoskeletal pain and disability [2]. Although the projected increase in the proportion of older people in the population has propelled OA up the agenda of health planners and policy makers, the main focus of attention has been on lower limb OA. Less attention has been given to the hand, despite the fact that the prevalence of hand pain in the general population has been estimated between 12% and 21% [3-5] and at older ages the hand is one of the most common sites of pain and OA [6]. The relationship between symptomatic hand OA and disability in everyday life has not been studied in detail [7], and although there is some evidence that older people with hand problems suffer significant pain and disability [8] and psychological and emotional distress as a result of functional limitation [9], little is known about the specific ways in which these problems interfere with daily life, or how their impact varies with age, gender and pain severity. Although older people with hand problems view OA as a serious condition, the majority do not consult their general practitioner with their hand problem over the course of a year, even when severely affected [8].

Defining hand OA for epidemiological research and in clinical practice is problematic. Clinical criteria [10] and radiographic grading [11] for the classification of hand OA have been developed to establish uniformity in the reporting of this disease. However, population studies have shown that symptoms are only present in a minority of those with radiographic changes [12], suggesting that the clinical syndrome and the structural disease of OA appear to be separate, albeit related, entities. Consequently, it is doubtful whether the "true" prevalence of symptomatic hand OA can be captured from clinical or radiographic studies alone [10].

In North Staffordshire a programme of research into osteoarthritis in primary care is being undertaken. The programme comprises a series of linked studies designed to establish the optimal management of osteoarthritis in older adults in primary care. The clinical assessment studies are part of this programme and are prospective cohort studies whose main objective is to provide population-based evidence that will indicate the most useful way of assessing older adults with hand pain and problems and knee pain in primary care. The studies will provide primary care practitioners with a description of the population of older adults with hand pain and problems and knee pain in clinically meaningful terms i.e. using simple clinical history and examination techniques. Additionally, they should help to determine if clinical classification of musculoskeletal hand and knee conditions is useful at the population level and what simple questions and assessment tools identify important groups, both cross-sectionally and longitudinally. The aim of this paper is to outline the protocol for the Clinical Assessment Study of the Hand (CAS-HA). The protocol for the Clinical Assessment Study (Knee) (CAS(K)) has been reported previously [13].

### Cross sectional study

The general aim of the cross sectional component of the CAS-HA is to provide population-based evidence that will indicate the most useful way of assessing older adults with hand pain or hand problems in primary care. Additionally, we aim to identify clinical, functional and radiographic sub-groups within the study population. Specifically our study will consider the following questions:

• What is the prevalence of clinical signs and symptoms? How does this relate to hand function?

• What is the prevalence of 'red flags' indicative of possible serious joint pathology?

• In what respect do consulters and non-consulters differ at baseline?

• Can simple signs and symptoms accurately identify older adults with radiographic hand OA?

• What is the relationship between symptomatic hand OA and soft tissue syndromes e.g. carpal tunnel syndrome?

### Longitudinal study

Accurate information on the likely course of hand pain and problems in this population will play an important role in deciding how best to manage these problems and may possibly help to inform preventative measures in the future. To address this we intend to establish a cohort at baseline that will be followed up at 18-month intervals (subject to further funding and ethical approval). The study is designed in accordance with previously published requirements for reporting longitudinal studies in rheumatology [14,15]. The general aim of the longitudinal component of the CAS-HA is to determine the course of hand pain and problems over time. Specifically, our study will address the following questions:

• How common is deterioration in terms of hand pain, hand problems and functional limitation? Can this be predicted?

• Does radiographic OA predict change in severity and characteristics of symptomatic hand OA?

• What proportion of this sample consult their general practitioner for hand pain or problems within the follow-up period? Can this be predicted by information collected at baseline?

• What is the relative contribution of clinical history, hand assessment, digital imaging, x-rays and lower limb function as prognostic markers?

## Methods/Design

**Figure 1:**
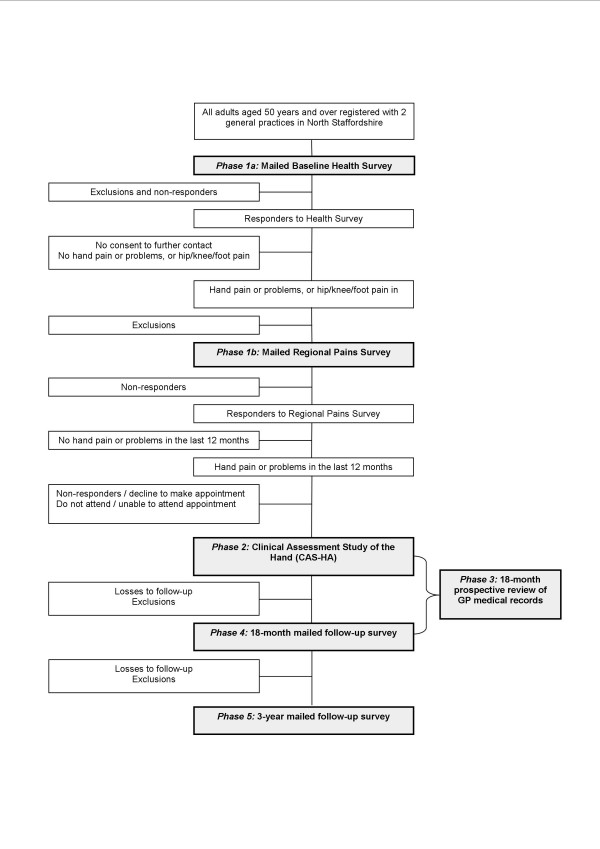
Flowchart of study procedures. Data collection points are in shaded boxes.

A population-based prospective observational cohort study of hand pain and problems in older people (50 years and over) has been designed in parallel to our previously reported cohort study of knee pain in older people [13]. The hand cohort study will be conducted in 5 phases with a sample of people, aged 50 years and over, registered with two local general practices (Figure 1). Ethical approval for CAS-HA baseline and 18-month follow up has been obtained from the North Staffordshire Local Research Ethics Committee. Ethical approval for 3-year follow up has been obtained from the Hereford and Worcester Local Research Ethics Committee.

*Phase 1: *Baseline two-stage mailed survey

*Phase 2: *Baseline clinical assessment study of the hand (CAS-HA)

*Phase 3: *Eighteen month prospective review of general practice medical records

*Phase 4: *Follow-up mailed survey at 18 months

*Phase 5: *Follow-up mailed survey at 3 years

### Phase 1: Baseline two-stage mailed survey

Full details of Phase 1 design and methods have been previously reported [16]. Briefly, Phase 1 consists of a Health Survey questionnaire that will be mailed to all adults aged 50 years and over registered with the two participating practices. Respondents who provide written consent to further contact and who report pain or problems (e.g. stiffness or knobbly swellings) in the hands, or pain in the hips, knees or feet will be sent a second questionnaire (the Regional Pains Survey questionnaire). These two questionnaires include measures of general health status, socio-demographic characteristics, psychological and lifestyle variables, and pain and disability (general and site specific). Hand specific questions are provided in Table 1. Non-responders to each questionnaire will be sent a reminder postcard at two weeks and, for those who do not respond to the postcard, a repeat questionnaire at 4 weeks.

**Table 1:** Hand specific data to be collected at baseline (Regional Pains Survey Questionnaire)

**Concept**	**Measurement method**	**Details**
Characteristic of complaint	Hand dominance	right, left, both
	Duration of hand problem	years/months
	Hand problem in past 12 months*^§^	yes, no
	Hand pain in past 12 months*^§^	yes, no
	Side of pain in past 12 months*^§^	right, left, both
	Duration of pain in past 12 months*^§^	< 7 days, 1–4 weeks, 1–3 months, 3+months
	Most problematic hand*^§^	right, left, both
Hand pain, symptoms and physical features	AIMS 2*^§ ^[30]	pain sub-scale
	AUSCAN*^§ ^[34]	pain and stiffness sub-scales
	In past month, severity of stiffness, aching, tenderness, weakness, clumsiness, burning, tingling, numbness *^§^	severe, moderate, mild, very mild, none
	In past month, days of joint warmth, dropping objects, frustration *^§^	all, most, some, few, no
	Hand pain lasting ≥ 1 day in past month*^§‡^	yes, no
	Painful areas in last month: hand drawings [31]*^§^	shaded areas
	Nodes: hand drawings*^§ ^[32]	circled joints
Aesthetics	Michigan Hand Outcomes Questionnaire^§ ^[33]	appearance sub-scale
Function	AIMS 2*^§‡ ^[30]	hand and finger function sub-scale
	AIMS 2^§ ^[30]	arm function sub-scale
	AUSCAN*^§ ^[34]	physical function sub-scale
	Difficulty with usual activities: pick up coins, hold book, clench fist, self-care, open packets	no, mild, moderate, severe, unable to do
Illness perceptions	Illness Perceptions Questionnaire Revised (IPQ-R) [35]	9 dimensions: illness coherence, treatment control, personal control, timeline (acute/chronic), timeline (cyclical), consequences, emotional representation, identity, causes
Health care related to hand problem	AIMS 2*^§ ^[30]	medication sub-scale
	Hand injuries ... ever	yes, no: right, left, both
	Hand operations ... ever	yes, no: right, left, both
	Consulted GP in past 12 months^§^	yes, no
	NHS and private services used in past 12 months^§ ^(adapted from [36])	yes, no to physiotherapy, occupational therapy, hospital specialist, acupuncture, osteopath/chiropractor, drugs on prescription, hand operation, hand injection, other
Occupational impact	Excessive use of hands in occupation	yes, no
Pastimes and hobbies	Excessive use of hands in pastimes and hobbies	yes, no
Impact of symptoms	AIMS 2*^§ ^[30]	impact subscale

*Also gathered at 18 months; ^§ ^Also gathered at 3 years; ^‡ ^Minimum data to be sought at 18 months and 3 years from non-responders

### Phase 2: Baseline clinical assessment study of the hand (CAS-HA)

Respondents to the Regional Pains Survey questionnaire who report experiencing hand pain or problems within the last 12 months and who provide written consent to further contact will be sent a letter of invitation to the CAS-HA research clinic and an information sheet outlining the study. The process of recruiting participants and the practical organisation and running of the CAS-HA research clinic will follow the same procedures as those reported previously for CAS(K) [13]. Briefly, participants will be offered an appointment to attend the research clinic where they will be assessed by a trained research therapist after giving written, informed consent. Research clinics will be held at a local National Health Service Trust Hospital and will offer a maximum of 16 appointments per week.

Participants will undertake the following standardised assessment: digital photography of the hands, clinical interview and hand assessment, lower extremity function test, brief self-complete questionnaire, plain radiography of the hands and knees, and simple anthropometric measurement.

#### Digital photography of the hands

Each participant will have four photographs taken of their hands by an assessor using a digital camera (Olympus Camedia C-4040 ZOOM: resolution 2272 × 1704 pixels) attached to a copy stand. The dorsal and palmar aspects of both hands, including the wrists, will be photographed. Photographs will be taken according to pre-defined written protocols that include standard positioning of participants.

#### Clinical interview and hand assessment

Participants will be interviewed and examined by a trained assessor blinded to the findings from radiography and digital photography. The proposed content of the interview and assessment is provided in Table 2. Briefly, this procedure will comprise two components. Firstly, participants will be screened to identify possible red flags indicative of potentially serious pathology, namely recent trauma to the hands likely to have resulted in significant tissue damage, and acutely swollen, painful hands or knees. Secondly, a structured, standardised clinical interview and hand assessment developed and piloted for the study will be conducted [17,18]. For assessments requiring instrumented measures, equipment will be calibrated prior to the start of the study.

**Table 2:** Hand specific data to be collected during clinical assessment (CAS-HA)

**Concept**	**Measurement method**	**Details**
*Clinical Interview Questions:*		

Characteristic of complaint	Duration of hand problem	< 12 months, 1-<5 years, 5-<10 years, 10 years +
	Onset: sudden, gradual	yes, no, for right and left hands
	Onset: following accident or injury	yes, no, for right and left hands
Hand pain and hand symptoms	Pain/tenderness in past month	yes, no
	Hand pain descriptors from McGill Pain Questionnaire^§ ^[37]	15 descriptors
	Pain location: hand drawing	shading both hands front and back
	Pain present all the time	yes, no
	Pain related to sleep disturbance	yes, no
	Pain limits activity	yes, no
	Hand stiffness in past month	yes, no
	Side of stiffness	right, left, both
	Hand stiffness on waking in past month	yes, no
	Duration of morning stiffness	≤ 30 mins, 30+ mins
	Finger locking, triggering	yes, no
	Release of locking	yes, no
	Altered sensation (pins + needles, tingling, numbness) in past month	yes, no
	Altered sensation location: hand drawing	shading both hands front and back
	Altered sensation worse at night	yes, no
Occupational impact	Stop work due to hand problem	yes, no
	Absence from work due to hand problem	yes, no
Management/self-help	Adaptation: gadgets, help, avoidance, change method, stop/reduce activity, take longer, other	yes, no
	17 treatments/self-help activities tried recently	yes, no
	Any treatments effective	yes, no
Family history of joint problems	Relatives with joint problems: father, mother, brother, sister	yes, no
	Hand involvement	yes, no
Diagnostic and causal attributions	Open-ended questions	free text
Health problems	Open-ended question: 2 most important health problems	free text

*Hand Assessment (right and left hands):*		

Upper limb screen	9 movements (adapted from [38])	yes, no, unable to assess
Observation/Palpation	Swelling, nodes, bony enlargement, deformity at selected joints	yes, no
	Thenar muscle wasting	yes, no
	Dupuytren's	yes, no
Measurement	Thumb opposition [39]	yes, no, for 10 positions
	Thumb extension	degrees
	Wrist extension	degrees
	Wrist flexion	degrees
Tests	Phalen's [40,41]	positive, negative, unable to assess
	Grind [42,43]	positive, negative, unable to assess
	Finklestein's [42,44]	positive, negative, unable to assess
Hand function	Grip Ability Test [45]	timed (seconds)
	Power grip (JAMAR dynomometer) [46]	lbs
	Pinch grip (B&L pinch gauge) [46]	lbs

*Brief self-complete questionnaire:*		

Hand pain and hand symptoms	Days of hand pain, ache or stiffness in past month*^§ ^[10]	all, most, some, few, no
	Severity of hand pain in past month*^§^	numerical rating scale (0–10)
	Thumb pain during activity in past month*^§^	yes, no
	Swelling in hands in past month	yes, no
Impact of symptoms	Severity of overall hand problems in past month*^§^	none, very mild, mild, moderate, severe
	Bothersomeness of hand problem in past 2 weeks*^§ ^(adapted from [47])	not at all, slightly, moderately, very much, extremely
	Symptom satisfaction*^§ ^(adapted from [23])	5-point Likert scale: very dissatisfied to very satisfied

*Also gathered at 18 months; ^§ ^Also gathered at 3 years; ^‡ ^Minimum data to be sought at 18 months and 3 years from non-responders

#### Lower extremity function

The Short Physical Performance Battery (SPPB) [19] will be conducted in all participants. This includes a standing balance test, a timed repeated chair stand test (5 repetitions) and a 4-metre gait speed test. The conduct and scoring of the SPPB will be as recommended on the training CD-ROM (Guralnik, personal communication).

#### Brief self-complete questionnaire

During the clinic visit, participants will complete a brief self-complete questionnaire containing questions relating to their hand problem (Table 2). Questions relating to knee problems will also be asked – days of pain, aching or stiffness in previous month, days in pain in the previous 6 months [20], episode duration [21], the Chronic Pain Grade [22] and symptom satisfaction (adapted from [23]).

#### Radiography and anthropometric measurement

Radiography of both hands and knees will be obtained for all participants. Plain radiographs of each hand will be taken (1 hand per film). A posteroanterior (PA) view will be taken, where the palmar aspect of the hand will be placed on the film with the fingers extended, separated slightly and spaced evenly (Buckland-Wright, personal communication). Imaging of the tibiofemoral joint of the knee will be undertaken using weight-bearing semiflexed (MTP) posteroanterior (PA) view according to a defined protocol [24]. The patellofemoral joint of the knee will be imaged with the lateral and skyline view, both in a recumbent position with the knee flexed to 45°. Weight (kgs) and height (cms) of each participant will be measured using digital scales (Seca Ltd., Birmingham, UK) and a wall mounted height meter (Holtain Ltd., Crymych, UK) respectively.

#### Post-clinic procedure

The practical organisation, administration and communication post-clinic will be identical to that described by Peat et al [13], but with emphasis on the hand rather than the knee. A trained observer with a background in diagnostic radiography will score the hand radiographs. Standardised coding of radiographic features using the Kellgren and Lawrence [11] grading system will be completed for sixteen joints in each hand and wrist, the distal interphalangeal joints (DIP), the proximal interphalangeal joints (PIP), the interphalangeal joint of the thumb (IP), the metacarpophalangeal joints (MCP), the thumb carpometacarpal joint (CMC) and the trapezioscaphoid joint (TS). Knee films will be scored for individual radiographic features, including osteophytes, joint space narrowing, sclerosis and subluxation. The Altman Atlas [25] and scoring system [26] are to be used for the PA and skyline views and the Burnett Atlas [27] for the lateral view. Additionally, PA and skyline views will be assigned a Kellgren and Lawrence grade [11].

#### Quality assurance and quality control

Quality assurance and control are important in longitudinal studies especially when using observers to gather data [28]. In the current study, the clinical interview, hand assessment, lower limb screen, and the taking and scoring of radiographs will be subject to a number of quality assurance and control procedures.

The study protocol and inter- and intra-assessor reliability of the clinical interview and hand assessment have been formally tested in a pilot study [18]. Reliability studies investigating inter- and intra-observer reproducibility will be conducted for the scoring of radiographs.

All assessors will receive training using the study protocols prior to the commencement of data collection. Assessors will practice interviews and assessments using the protocols with healthy volunteers and expert participants. All radiographers participating in the study will also receive training prior to the start of the research clinics. A detailed assessor manual containing study protocols will be provided to all members of the CAS-HA team for reference during the study period. A programme of quality control measures previously reported [13] will be implemented throughout the course of the study.

### Phase 3: Prospective review of general practice medical records

All participants in Phase 1 who give written consent for their GP records to be accessed will have their computerised medical records tagged by a member of the Centre's Health Informatics team. The protocol for this phase of the study has been previously reported [13].

### Phase 4 and 5: Follow-up mailed survey at 18 months and 3 years

A follow-up survey will be mailed to all Phase 2 participants 18 months and 3 years after their baseline clinical assessment. The focus of follow-up will be on clinical change in symptoms and function and possible determinants of this. The proposed content of these surveys is provided in Tables 1, 2, 3. Primary outcome data will be sought from non-respondents by telephone or post. Participants who have moved practice during the follow-up period will be traced using the NHS tracing service and their new general practitioner will be asked for permission to include them in the follow-up.

**Table 3:** Hand specific data to be collected only at 18 months and 3 years

**Concept**	**Measurement method**	**Details**
Perceived change in hand problem since baseline	Transition index [48]^‡^	completely recovered, much better, better, no change, worse, much worse
Health care related to hand problem since baseline	Hand injury	yes, no
	Hand operation	yes, no
	Consulted GP in past 18 months^†^	yes, no
	NHS and private services used in past 18 months^† ^(adapted from [36])	yes, no to physiotherapy, occupational therapy, hospital specialist, acupuncture, osteopath/chiropractor, drugs on prescription, hand operation, hand injection, other
Occupational impact since baseline	Time off work	yes, no
	Stopped work	yes, no
Hand pain and hand symptoms	Days of hand swelling in past month	all, most, some, few, no
	Days of hand pain in past 6 months^ [22]	no, 1–30, 31–89, 90+ days
	Hand pain severity in past 6 months^	numerical rating scale (1–10)
Coping strategies for hand pain	Single-item Coping Strategies Questionnaire (CSQ) [49]	numerical rating scale (0–7) with verbal anchors (never do that, always do that)
Illness perceptions	Shortened version adapted from IPQ-R [35]	6 dimensions: illness coherence, personal control, timeline (acute/chronic), timeline (cyclical), consequences, emotional representation
Management/self-help	7 treatments/self-help activities tried in past month: simple painkiller; anti-inflammatory tablets; creams, gels, or rubs; glucosamine or chondroitin sulphate; warmth, heat; cold; hand exercises	yes, no
Narrative account	Open-ended question: course of hand pain and problems^‡^	free text

^‡ ^Minimum data to be sought at 18 months and 3 years from non-responders; ^†^Data only gathered at 18 months; ^Data only gathered at 3 years

### Sample size

The sample size for this study was determined by the estimated numbers of participants needed in Phase 2 to ensure sufficient power for both cross-sectional and longitudinal analyses. A target sample of 500 was set. We estimate that 90% of follow-up questionnaires will be returned and that approximately 70 participants (12%) will report clinically significant deterioration over the 18-month period [29]. With this number of participants, we will have 80% power to detect a risk ratio of 1.6 or greater with a minimum 64% exposure rate (e.g. presence of radiographic OA) in those who have deteriorated, and a 50% exposure rate in those who do not, at 95% level of confidence.

### Statistical analysis

Linking data collected at the clinical assessment with that from the 18-month and 3-year follow-up questionnaires, we will be able to determine prospectively the factors that are related to clinical deterioration using risk ratios and associated 95% confidence intervals.

## Discussion

The Clinical Assessment Study of the Hand (CAS-HA) is a prospective, population-based, observational cohort study based in North Staffordshire that intends to investigate issues surrounding the classification and course of hand pain, problems and hand osteoarthritis in community-dwelling adults aged 50 years and over. This study will complement our previous study on knee pain in older people [13].

## Abbreviations

AIMS2, Arthritis Impact Measurement Scale 2; AUSCAN, AUStralian CANadian Osteoarthritis Hand Index; CAS-HA, Clinical Assessment Study of the Hand; CAS(K), Clinical Assessment Study of the Knee; CMC, carpometacarpal; CSQ, Coping Strategies Questionnaire; DIP, distal interphalangeal; GP, General Practitioner; IP, interphalangeal; IPQ-R, Illness Perceptions Questionnaire Revised; MCP, metacarpophalangeal; MTP, metatarsophalangeal; OA, Osteoarthritis; PA, posteroanterior; PIP, proximal interphalangeal; SPPB, Short Physical Performance Battery; TS, trapezioscaphoid.

## Competing interests

The author(s) declare that they have no competing interests.

## Authors' contributions

All authors participated in the design of the study and drafting the manuscript. All authors read and approved the final manuscript.

## Pre-publication history

The pre-publication history for this paper can be accessed here:


